# The impact of trauma and daily hardships on the mental health of unaccompanied refugee minors detained in Libya

**DOI:** 10.1192/bjo.2022.622

**Published:** 2023-01-05

**Authors:** Ilse Derluyn, Giacomo Orsini, Floor Verhaeghe, Rihab Elhaj, Ine Lietaert, Elisa Pfeiffer

**Affiliations:** Department of Social Work and Social Pedagogy, Centre for the Social Study of Migration and Refugees, Faculty of Psychology and Educational Sciences, Ghent University, Ghent, Belgium; Department of Social Work and Social Pedagogy, Centre for the Social Study of Migration and Refugees, Faculty of Psychology and Educational Sciences, Ghent University, Ghent, Belgium; and UNU-CRIS, Brugge, Belgium; Clinic for Child and Adolescent Psychiatry/Psychotherapy, Ulm University, Ulm, Germany; DAI, Bethesda, Maryland, USA

**Keywords:** Trauma, post-traumatic stress disorder, transcultural psychiatry, low- and middle-income countries, childhood experience

## Abstract

**Background:**

The high trauma load and prevalence of mental distress in unaccompanied refugee minors (URMs) who resettle in Western (European) countries is well documented. However, the lack of studies investigating the potentially most vulnerable population, URMs who are currently on the move in transit countries such as Libya, is alarming.

**Aims:**

To document the mental health of URMs detained in Libya and the possible associations with trauma, flight and daily hardships.

**Method:**

In total *n* = 99 (94.9% male; *n* = 93) URMs participated in this cross-sectional study conducted in four detention centres near the Libyan capital Tripoli. Data were collected via standardised questionnaires in an interview format and analysed using structured equation modelling.

**Results:**

Participants reported high rates of trauma, especially within Libya itself. Reports of daily hardships in detention ranged between 40 and 95% for basic needs and between 27 and 80% for social needs. Higher social needs were associated with increased anxiety symptoms (*β* = 0.59; *P* = 0.028) and increased pre-migration (*β* = 0.10; *P* = 0.061) and peri-migration trauma (*β* = 0.16; *P* = 0.017) with symptoms of depression. Similarly, higher levels of pre-migration trauma were associated with higher post-traumatic stress disorder levels (*β* = 0.17; *P* = 0.010).

**Conclusions:**

The rates of daily hardships and traumatic events are higher compared with those recorded for URMs living in asylum centres in Europe. The emotional, social and cognitive development of detained URMs is severely threatened in both the short and long term. This paper outlines some of the most detrimental effects of migration policies on URMs transiting through Libya.

The harsh conditions that refugees and migrants face as they transit through war-torn Libya en route to Europe are well-documented, in particular through reports from international non-governmental organisations (NGOs).^1^ According to the findings of the United Nations’ (UN) Panel of Experts on Libya, appointed by the UN Security Council, migrants and asylum applicants in Libya suffer ‘violations of [their] human rights, including kidnappings, arbitrary detentions and summary executions’.^2^ Owing to bi- and multilateral agreements that Libya has signed with the European Union (EU) and its single member states, migrants and refugees transiting through the country are subject to restrictive policies, including detention. Importantly, such policies are implemented by law enforcement officials who often have close connections with local militias and smuggling or trafficking networks. These policies also apply to unaccompanied refugee minors (URMs), minors migrating without their parents; as a consequence, URMs often suffer extreme consequences from transiting through this country.^3^ A large body of research suggests that URMs in the peri-migration period report alarmingly high rates of traumatic experiences and daily hardships, and consequently elevated psychological distress, including symptoms of post-traumatic stress disorder (PTSD), depression and/or anxiety.^4^ Adult refugees and children in migrant detention centres demonstrate even higher rates of mental and physical health problems.^5,6^ Existing research generally focuses on retrospective reporting by URMs who are already in Europe and who were detained months, if not years, earlier^7^ or it provides analyses of the cases of refugee minors who were detained in immigration centres located in non-European Western countries, such as Australia^5,8^ or the USA.^9^ Although there is a growing evidence base on the detrimental impact of detention on adult refugees,^10^ despite the fact that the detention of URMs is increasing worldwide,^5^ we still lack studies on the mental health of detained URMs, especially those still in transit to another country.

## Aims

This study documents the mental health of URMs who are currently in detention centres in Libya, a frequent transit country for refugees on their way to Europe. Furthermore, this study aims to investigate how URMs’ mental health is associated with trauma prior to and during flight, as well as the daily hardships they experience during detention. Because data were gathered in Libyan migrant detention centres, this paper constitutes an exploratory study whose overarching goal is to expand the existing understanding of the mental health of URMs by focusing on the subset emigrating to Europe through the North African country of Libya.

## Method

This study is part of the European Research Council (Horizon project number: 714222) funded project ChildMove, a mixed-methods (quantitative and qualitative data), longitudinal and multi-country (Libya, Italy, Greece, Belgium) study. In this paper, we focus on the quantitative data collected in Libya which, where relevant, are corroborated by further insights from the qualitative data-set.

### Ethics statement

The authors assert that all procedures contributing to this work comply with the ethical standards of the relevant national and institutional committees on human experimentation and with the Helsinki Declaration of 1975, as revised in 2008. All procedures involving human participants were approved by the Institutional Review Board at Ghent University, Faculty of Psychology and Educational Sciences (#2017-23-Ine Lietaert). All study participants gave their written informed consent before being enrolled in the study.

### Recruitment and study setting

Data were collected between April and July 2018 in four detention centres located in the surroundings of the Libyan capital Tripoli. This means that all participants had been detained by authorities in facilities managed by the Government of National Accord (GNA) on behalf of the EU. The selection of the main researcher working in Libya was made by snowball sampling from a number of experts and members of relevant NGOs and international organisations, which operated as gatekeepers.

On the ground, the main researcher was supported by two other researchers. They were granted access to the different detention centres by obtaining official permission from members of the GNA and its administration – for example the Department for Combating Illegal Immigration. The research team was also in contact with, and received support from, the EU delegation in Libya.

### Participants

The study sample comprises *n* = 99 URMs (94.9% male; *n* = 93); see Table 1 for more information. Originally, we intended to collect data from 100 minors in order to have a sufficiently large and representative sample of the population of URMs detained in Libya. However, by the end of the time frame of the official permission, we could only interview 99 participants.

**Table 1:** Sociodemographic characteristics of the study participants (*n* = 99)

Characteristic	
Gender, *n* (%)	
Male	93 (94.9)
Female	6 (5.1)
Country of origin *n* (%)	
Chad	1 (1)
Cote d‘Ivoire	2 (2)
Eritrea	50 (50.5)
Ethiopia	1 (1)
Liberia	1 (1)
Mali	2 (2)
Nigeria	5 (5)
Senegal	1 (1)
Sierra Leone	2 (2)
Somalia	26 (26.3)
Sudan	5 (5)
Living situation, *n* (%)	
Detention centre together with adults	98 (99)
In an office^a^	1 (1)
Average flight duration, mean (s.d.; range)	22.7 months (11.4; 7–78)

a. This participant was held in a detention centre twice, but at the time of the study, he was living in a room that was used as an office.

### Measures

All measures were translated into 13 languages (either already available or translated–back-translated in this study) and assessed by trained assessors. Interpreters and cultural mediators were employed if the bi-/multi-lingual researcher and participant did not speak a common language. Next to the assessment of standardised measures, semi-structured qualitative interviews provided more in-depth insights into the living conditions experienced by URMs in Libya (beginning from time of entry into the country, thus capturing experiences both prior to and during detention). We included selected quotes from these qualitative data to enrich the quantitative findings and to give voice to URMs expressing their more personal and subjective feelings in their own words. For more detailed information on the qualitative methods, see Orsini et al (2022).^11^

#### Traumatic experiences

The Stressful Live Events (SLE) questionnaire^12^ is a self-report measure assessing 10 different potentially traumatic events (yes/no) at three time points: pre-migration, peri-migration and since the arrival in the current host country (Libya).

#### Daily stressors/hardships

The Daily Stressors Scale for Young Refugees (DSSYR)^13^ is a self-report measure consisting of 20 potential daily stressors/hardships, assessing to what extent these have been experienced during the past month on a 4-point Likert scale ranging from ‘never’ (1) to ‘always’ (4). Participants could also answer ‘I don't know/don't want to answer’, which is why the sample size of participants selecting answer options 1–4 is reported in Table 2 for each item. A recent (unpublished) validation study identified two subscales: ‘stress due to insufficient fulfilment of (basic) needs’ (‘basic needs’; 9 items; maximum score 36) and ‘stress due to social needs’ (‘social needs’; 6 items; maximum score 24) (Table 2). For more information on the subscales, see Behrendt et al (2022).^14^

**Table 2:** Frequency of reported daily stressors in *n* = 99 study participants

Daily stressors^a^	Never, *n* (%)	Sometimes, *n* (%)	Often, *n* (%)	Always, *n* (%)	Total ‘yes’,^b^ *n* (%)
Basic needs					
Not enough food (*n* = 98)	15 (15.3)	27 (27.6)	15 (15.3)	41 (41.8)	83 (85.7)
Not enough clothing (*n* = 97)	5 (5.2)	14 (14.4)	16 (16.5)	62 (63.9)	92 (95.8)
Not enough money (*n* = 96)	20 (20.8)	8 (8.3)	8 (8.3)	60 (62.5)	76 (79.2)
Not enough housing (*n* = 98)	19 (19.4)	21 (21.4)	15 (15.3)	43 (43.9)	79 (80.6)
Not enough medical care (*n* = 98)	30 (30.6)	26 (26.5)	13 (13.3)	29 (29.6)	68 (69.4)
Not enough education (*n* = 95)	28 (29.5)	5 (5.3)	8 (8.4)	54 (56.8)	67 (70.5)
Lack of information (on procedures, rights etc.) (*n* = 94)	22 (23.4)	14 (14.9)	13 (13.8)	45 (47.9)	72 (76.6)
Feelings of unsafety (*n* = 97)	30 (30.9)	35 (36.1)	7 (7.2)	25 (25.8)	67 (69.1)
Being forcibly and repeatedly moved (*n* = 96)	58 (60.4)	16 (16.7)	11 (11.5)	11 (11.5)	38 (39.6)
Social needs					
Difficulties in making new friends (*n* = 98)	57 (58.2)	15 (15.3)	13 (13.3)	13 (13.3)	41 (41.8)
Difficulties in communicating with others due to the foreign language (*n* = 96)	22 (22.9)	27 (28.1)	15 (15.6)	32 (33.3)	74 (77.1)
Hearing people say bad things about myself (*n* = 97)	71 (73.2)	21 (21.6)	2 (2.1)	3 (3.1)	26 (26.8)
Feeling bored (*n* = 97)	20 (20.6)	40 (41.2)	16 (16.5)	21 (21.6)	77 (79.4)
Feeling of being treated unfairly compared with others (*n* = 98)	58 (59.2)	21 (21.4)	9 (9.2)	10 (10.2)	40 (40.8)
Feeling that others have prejudices about myself or people of my country/culture (*n* = 96)	61 (63.5)	19 (19.8)	6 (6.3)	10 (10.4)	35 (36.5)
Other stressors					
Worrying about my family at home (*n* = 96)	5 (5.2)	13 (13.5)	20 (20.8)	58 (60.4)	91 (94.8)
Difficulties in obtaining legal documents (*n* = 69)	36 (52.2)	8 (11.6)	9 (13)	16 (23.2)	33 (47.8)
Difficulties related to the age-assessment procedures (*n* = 40)	26 (65)	8 (20)	1 (2.5)	5 (12.5)	14 (35)
Feeling uncertain about the future (*n* = 95)	15 (15.8)	31 (32.6)	18 (18.9)	31 (32.6)	80 (84.2)
Other difficulties that I experienced last month (*n* = 55)	39 (70.9)	8 (14.5)	2 (3.6)	6 (10.9)	16 (29.1)

a. The *n* is reported for each item individually as participants could also check ‘I don't know/don't want to answer’.

b. Number of participants who answered either ‘sometimes’, ‘often’ or ‘always’.

#### Post-traumatic stress disorder

The Reactions of Adolescents to Traumatic Stress (RATS) questionnaire^15^ is a multicultural self-report measure assessing the prevalence of PTSD symptoms according to DSM-IV criteria. For this study, a short version of the measure was used, as we aimed to make the assessment as short as possible owing to ethical considerations. Since this short version is not yet validated and no longer represents the full PTSD DSM-IV criteria, we decided to develop a short version based on ICD-11 (five items on intrusion, one item on avoidance, two items on hyperarousal), as all symptoms of the ICD-11 criteria were assessed. Further advantages of the version using an ICD-11 conceptualisation are that we now assess PTSD in line with our current understanding of the disorder and can compare the results with future studies that assess PTSD using ICD-11-based measures. The final eight items assess symptoms on a scale from ‘not’ (1) to ‘very much’ (4) (maximum score 32). The Cronbach's alpha in this study was 0.82.

#### Mental health

Symptoms of anxiety (10 items; maximum score 40) and depression (14 items; maximum score 56) were measured using the Hopkins Symptom Checklist-37 (HSCL-37A), which is a modified version of the HSCL-25 and specifically developed for URMs.^16^ The items are rated on a 4-point scale that ranges from ‘never’ (1) to ‘always’ (4). In the current study, the Cronbach's alphas were 0.80 for the anxiety subscale and 0.80 for depression.

### Statistical analysis

Descriptive statistics were conducted to describe sociodemographic characteristics, mental health scores and prevalence rates of potentially traumatic events and daily hardships. Missing data were replaced by employing multiple imputation with five imputed data-sets. The data were then analysed via structural equation modelling (SEM). The variables pre-migration trauma, peri-migration trauma and trauma in the host country, as well as daily hardship subscales (basic and social needs (latent variables)) and flight duration, served as predictors in a two-step model with the outcomes PTSD, anxiety and depression. All analyses were run using SPSS 26 and R Studio (package lavaan.mi) for Windows.

## Results

Participants reported high rates of trauma across all events and time points (Table 3), with a mean of 1.77 (s.d. = 1.93; range: 0–8) traumatic events pre-migration, a mean of 1.59 (s.d. = 1.86; range: 0–7) traumatic events peri-migration and a mean of 4.21 (s.d. = 2.02; range: 0–8) traumatic events since arrival in the current host country. The most prevalent of these traumatic events was witnessing physical abuse (*n* = 89; 89.9%), while the least prevalent was experiencing sexual abuse (*n* = 9; 9.1%). Besides events related to family or war, prevalence rates of events were highest in the current host country.

**Table 3:** Frequency of reported potential traumatic experiences/stressful life events in *n* = 99 study participants at different time points of their migration

Traumatic experience	No, *n* (%)	In my home country, *n* (%)	On my way to this country, *n* (%)	Since arrival in this country/place, *n* (%)	Total yes, *n* (%)
Drastic changes in family	55 (56.1)	30 (30.6)	7 (7.1)	12 (12.3)	43 (43.9)
Forced separation from family	67 (68.4)	20 (20.4)	7 (7.1)	5 (5.1)	31 (31.6)
War or armed military conflict	51 (51.5)	37 (37.3)	5 (5)	14 (14.1)	48 (48.5)
Forced labour/to work	42 (42.9)	14 (14.3)	5 (5.1)	41 (41.9)	56 (57.1)
Experienced physical violence	19 (19.2)	11 (11.2)	29 (29.4)	70 (70.9)	80 (80.8)
Witnessed physical violence	10 (10.1)	19 (19.1)	31 (31.3)	73 (73.7)	89 (89.9)
Experienced sexual violence	90 (90.9)	4 (4)	2 (2)	6 (6)	9 (9.1)
Detention or imprisonment	6 (6.1)	19 (19.4)	19 (19.3)	86 (87.8)	92 (93.9)
Other very stressful life event with great danger	21 (21.4)	14 (14.2)	34 (34.7)	58 (59.1)	77 (78.6)
Other very stressful life event with someone else in great danger	24 (24.5)	15 (15.2)	25 (25.5)	57 (58.1)	74 (75.5)

Reports of daily hardships in the detention centres were high, ranging between 40 and 96% for basic needs (mean 18.97; s.d. = 5.38) and between 27 and 80% for social needs (mean 6.06; s.d. = 1.68) (Table 2). The most prevalent was not having enough clothing (95.8%) and worries about family at home (94.8%). The least prevalent reported hardship was ‘hearing other people say bad things about myself’ (26.8%).

Levels of PTSD (mean 19.99; s.d. = 5.45; range: 8–32), anxiety (mean 19.05; s.d. = 4.82; range: 10–39) and depression (mean 30.93; s.d. = 7.72; range: 16–65) were relatively high.

For all SEM analysis, *n* = 1 participant was not considered because the participant did not complete the PTSD and daily hardships scales (the RATS and DSSYR). The fit indices of the two-step model were acceptable, with the comparative fit index (CFI) being just below the limit of 0.9 (Tucker–Lewis index TLI = 0.9128; CFI = 0.8947; root mean square error of approximation RMSEA = 0.0583). The results of the SEM are presented in Table 4. Higher social needs were significantly associated with symptoms of anxiety (*β* = 0.59; *P* = 0.028). Depression was significantly associated (but only marginally) with pre-migration (*β* = 0.10; *P* = 0.061) and peri-migration trauma (*β* = 0.16; *P* = 0.017), as increased trauma resulted in higher levels of depression. Similarly, higher amounts of pre-migration trauma were significantly associated with higher levels of PTSD (*β* = 0.17; *P* = 0.010).

**Table 4:** Results of structural equation modelling (*n* = 98)

	Estimate (*β*)	s.e.	*t*	d.f.	*P*
Post-traumatic stress disorder					
Pre-migration trauma	0.171	0.066	2.597	535.729	0.010
Peri-migration trauma	0.098	0.065	1.505	1880.106	0.132
Trauma in host country	0.044	0.062	0.721	5992.287	0.471
Daily stressors: basic needs	−0.032	0.326	−0.097	Inf.	0.923
Daily stressors: social needs	0.504	0.313	1.611	Inf.	0.107
Flight duration	0.008	0.008	1.023	29.299	0.315
Anxiety					
Pre-migration trauma	0.002	0.067	0.035	140.334	0.972
Peri-migration trauma	0.028	0.071	0.400	593.360	0.690
Trauma in host country	0.081	0.064	1.270	2145.278	0.204
Daily stressors: basic needs	−0.175	0.279	−0.626	860.826	0.532
Daily stressors: social needs	0.593	0.271	2.192	4430.354	0.028
Flight duration	0.011	0.009	1.284	43.560	0.206
Depression					
Pre-migration trauma	0.098	0.052	1.876	2394.892	0.061
Peri-migration trauma	0.161	0.067	2.398	604.036	0.017
Trauma in host country	0.004	0.059	0.071	7567.506	0.943
Daily stressors: basic needs	−0.134	0.319	−0.419	Inf.	0.675
Daily stressors: social needs	0.459	0.336	1.368	Inf.	0.171
Flight duration	−0.004	0.008	−0.498	155.228	0.619

Inf., positive and negative infinity.

## Discussion

This is the first study that investigates the mental health, traumatic experiences and daily hardships of URMs in detention in Libya. The data show that this vulnerable population reports high rates of trauma, with the highest number of events occurring in the current host country, Libya. Compared with studies investigating the mental health of URMs in different European countries (most of which use post-migration samples), rates of traumatic experiences in the current host country are higher in this sample.^17^ Experiencing and witnessing physical violence are among the most prevalent events in this cohort, which is in line with current research.^18^ Making matters worse, URMs often rely on the perpetrators of physical violence in order to cross the country, as reported by this 19-year-old interviewee from Eritrea: ‘From [the Libyan city of] Sabratha we kept changing cars. From one small car to [ … ] another. [ … ] When we arrived in Sabratha [the smugglers] put us in a small room. [After they took us to the sea the] boat started sinking. [ … ] They took us back to the same room and started beating us’.^19^ Violence continues when URMs fall under the control of Libyan authorities; national law enforcement agencies often work in coordination with smuggling networks, as confirmed in the testimony of this 16-year-old URM: ‘When we were in prison a policeman came with smugglers: we were five, three of us were women and two of them were pregnant. They beat and raped them, all of them died and only me with another man [survived so] they asked us to pay 2000 dollars [ … ] When we [told them] that we [did not] have any money they started beating us’. Importantly, based on the experience of this young study participant detained in a government facility in the outskirts of Tripoli, the presence and work of international organisations such as the United Nations High Commissioner for Refugees (UNHCR) and the International Organization for Migration (IOM) – who at the time of the interview were in charge of providing social and healthcare services in the centre – is not enough to guarantee URMs’ safety: ‘[in the centre] we live with not enough food or water and [the guards] beat us every day, and they only care about money: I feel like I am still living with smugglers, not with the UNHCR’.

Specific traumatic events, such as imprisonment or forced labour, which are often related to the Libyan context, are higher in this study. Apparently, in 2018, the city of Bani Waled, located roughly 180 km south-east of Tripoli, functioned as a sort of hub where those who had just crossed the Sahara had to wait, hidden in informal detention facilities, before attempting the Mediterranean crossing. Almost all of the URMs interviewed who had spent time there told us that they were forced to work, as reported by this 17-year-old participant: ‘In Bani Waled, we worked against our will … They forced us to … We worked carrying rocks in house building [ … ] and they did not pay’.

The rates of reported daily hardships are dramatically higher when compared with the results of similar studies on daily hardships experienced by URMs in open asylum centres in Europe. One example is the lack of fulfilment of basic needs, which is reported by only 16.7–44.4% of participants in Belgium,^20^ whereas in Libya it is noted by 40–95% of the sample. Children and adolescents who face such high numbers of daily hardships are severely threatened in their emotional, social and cognitive development.^20^

Rates of mental health difficulties are also high in this sample. This is confirmed by the growing body of research on high prevalence rates among URMs who have resettled in high-income^21^ as well as low- or middle-income countries^22^ and among refugees who have been or currently are in detention.^7,8,10,23^ In our study, levels of PTSD and depression were significantly associated with traumatic experiences in the pre- and peri-migration periods, which is in line with studies on adult refugees detained in Australia.^8^ This can be explained by the well-established dose–response relationship, in which an increase in trauma results in a higher symptom load and has been demonstrated repeatedly in studies with URMs.^24^ Trauma in the host country was not associated with mental health problems, which could be explained by several factors, including the limited sample size. Another possible explanation could be that the participants were experiencing trauma – their stay in detention – at the time of the interview and were simultaneously aware of the further difficult experiences they would face post-detention as they attempted to reach the Libyan coast and cross the Mediterranean. Thus, while they could hardly process the traumas they were still experiencing, they also knew that mental strength would be necessary to successfully reach Europe – i.e. they had to stay in survival mode.^25^ In total, *N* = 44 participants revealed that since entering Libya they had tried to cross the Mediterranean at least once, only to be detected by the Libyan Coastguard and brought back to detention. Through this process, these URMs somehow came to ‘normalise’ the harsh conditions they faced daily in Libya. In this respect, the words of this 16-year-old URM interviewed in Belgium (data from the ChildMove project) after they had successfully crossed the Mediterranean are revealing: ‘[In Libya] there was no food, there was punishment … I did not have money. They did not believe me … so [that] they have to kick me a lot … and [there was no] food … But it is normal … it is normal life in Libya’.^11^

Regarding daily hardships, only social needs were associated with increased anxiety. Daily worries about family and friends and other social hardships, such as lack of friends or being confronted with prejudice and discrimination, may feed directly into a constant level of stress (physiological anxiety) and dysfunctional anxiety-related cognition, such as ‘the world is a dangerous place’ or ‘everyone could hurt me’, both of which are known to maintain symptoms.^26^

Moreover, being isolated from family members in detention is associated with more severe mental health symptoms, such as anxiety, in refugees.^8^ URMs, who by definition travel without their families and generally have only limited family contact (if at all) while in detention, lack the support from close family members while experiencing these unhealthy living situations; for this reason, they are particularly susceptible to developing even higher rates of mental distress than adult refugees. In our sample, almost all participants indicated that they worry about their families: 44% reported drastic familial changes and 32% noted forced separation from their families. This is a severe burden for any child or adolescent, and all the more serious for young refugees in incredibly difficult circumstances who have often left their families behind in challenging living conditions.

The level of daily hardships was not associated with depression or with PTSD, which can be explained by a potential ceiling effect. The URMs’ extremely high levels of social and basic needs might have resulted in small variations between participants and thus a smaller incremental effect on mental health. It is important to note, however, that mental health difficulties do not result from a single cause but from complex causal chains,^27^ of which trauma and daily hardships can only be considered parts. Several other established aetiological factors for depression, anxiety and PTSD must also be taken into consideration in future research: these include biological factors (e.g. genetic, neurological or hormonal mechanisms) and other environmental and personal vulnerabilities (stress–diathesis models including cognitive, interpersonal and personality factors).

### Limitations and future research

This study has several major strengths, including a relatively large sample, investigation of an extremely hard-to-reach but also vulnerable population and data collection carried out via both quantitative and qualitative instruments. However, several limitations should be considered. First, the gender imbalance of the study participants results in a limited generalisability of our findings to female URMs. Second, several participants (*n* = 6) stated in the trauma scale that they had not been imprisoned, which raises questions as to their overall understanding of the measure and/or their ability to respond freely to and/or trust the interviewer in a controlled environment such as the detention facilities visited. Third, although the RATS questionnaire has sound psychometric properties in its original version and is widely used with refugee adolescents,^15^ the scale itself is rather outdated and the ICD-11 version used in our study was not validated. Fourth, the depression subscale of the HSCL-25 did not show satisfactory validity in a recent validation study,^28^ which is why results on depression should be interpreted with caution. Fifth, this study included only cross-sectional data, which means that the analysis does not account for time; however, other studies of adult refugees have demonstrated an increase in symptoms the longer the individuals remained in detention.^8^ Future longitudinal studies with more heterogeneous samples might shed more light on URMs’ hardships and help generate findings concerning the (long-term) impact of trauma and daily hardships on this population's mental and physical health. The identification of risk and protective factors for the development of psychological distress can help develop adequate (preventive) interventions, which should be systematically evaluated and sustainably implemented. Finally, although the SLE is an established measure in the existing URM literature, it might not sufficiently address detention-specific traumatic events such as forced isolation from others or specific torture methods such as brainwashing.

### Clinical and political implications

This study exposes some of the most detrimental effects that current migration policies concerning entry into Europe can have on unaccompanied youth transiting in Libya. URMs are defined in European and UN official documents as a highly vulnerable population whose protection should be prioritised,^29^ yet our analysis of the outcomes produced by current migration management cooperation schemes between the North African country and the EU or individual European member states (e.g. Italy) suggests a different scenario. To externalise migration control to a war-torn country governed by a patchwork of militias and other criminal groups jeopardises the protection of young refugees by producing the *de facto* conditions for the systematic violations of their most fundamental rights. This study clearly demonstrates the detrimental effects of detention on the mental health of URMs and is therefore an urgent plea to end current detention practices.

## Data Availability

The data that support the findings of this study are available on request from the corresponding author (I.D.) The data are not publicly available owing to their sensitive nature. Additional related documents (study protocol and questionnaires, statistical analysis plan, informed consent) are also available on request from the corresponding author.
